# Supplementary material for: Dynamic transcriptomic profiles of zebrafish gills in response to zinc supplementation
Source: BMC Genomics. 2010 Oct 11;11:553. doi: 10.1186/1471-2164-11-553 (PMC3091702; doi:10.1186/1471-2164-11-553)
Supplement: Additional file 2 — Interactive Direct Interaction Network representing the molecular interactions between zinc, copper, iron, calcium and proteins encoded by transcripts changed by zinc supplementation. Mini web-site containing index.html and hyperlinked pages in subdirectory describing a Direct Interaction Network automatically generated based on curated interactions contained within the proprietary PathwayArchitect database. Ovals represent proteins and the circles symbolize metal ions. Objects are coloured by their abundance in zebrafish at the time-point they were significantly different from the control is a scale from -4 fold (dark green) to +4 fold (dark red). Where significant differences were found at more than one time-point, the colour overlay shows expression at the first instance. Dark blue squares denote 'binding', and light blue squares 'expression'; green squares stand for 'regulation', green diamonds for 'metabolism', and green circles for 'promoter binding'. Arrow heads indicate directionality of the interaction where annotated. All nodes and edges can be further interrogated by selecting the relative area of the image. [file 1471-2164-11-553-S2.zip › PathwayArchitect Zn xs DIN/156825.html]

# PROTEIN: POLR2D

|  |  |
| --- | --- |
| Name | POLR2D |
| Type | PROTEIN |
| Description | polymerase (RNA) II (DNA directed) polypeptide D |
| Note | This gene encodes the fourth largest subunit of RNA polymerase II, the polymerase responsible for synthesizing messenger RNA in eukaryotes. In yeast, this polymerase subunit is associated with the polymerase under suboptimal growth conditions and may have a stress protective role. A sequence for a ribosomal pseudogene is contained within the 3' untranslated region of the transcript from this gene. |
| Alias | DNA directed RNA polymerase II polypeptide D |
|  | POLR2D |
|  | 2310002G05Rik |
|  | RPB4 |
|  | HSRPB4 |
|  | HSRBP4 |
|  | RNA polymerase II subunit hsRBP4 |
|  | Polr2d |
|  | RBP4 |
|  | 2610028L19Rik |


---

|  |  |
| --- | --- |
| GO Component | nucleus |
|  | DNA-directed RNA polymerase II, core complex |


---

|  |  |
| --- | --- |
| GO ID | GO:0005665 |
|  | GO:0003899 |
|  | GO:0005634 |
|  | GO:0006350 |
|  | GO:0016779 |
|  | GO:0016740 |
|  | GO:0006366 |


---

|  |  |
| --- | --- |
| MIM | MIM:606017 |


---

|  |  |
| --- | --- |
| Connectivity | 21 |


---

|  |  |
| --- | --- |
| Entrez ID | 69241 |
|  | 5433 |


---

|  |  |
| --- | --- |
| Agilent ID | A\_14\_P130495 |
|  | A\_53\_P161515 |
|  | A\_51\_P419571 |
|  | A\_23\_P209778 |
|  | A\_32\_P219753 |


---

|  |  |
| --- | --- |
| Cellular Localization | Nucleus |
|  | Organelle |
|  | Cell |


---

|  |  |
| --- | --- |
| DbXref | Reactome##75071##mRNA Processing##http://www.reactome.org/cgi-bin/eventbrowser?DB=gk\_current&ID=75071 |
|  | Reactome##73894##DNA Repair##http://www.reactome.org/cgi-bin/eventbrowser?DB=gk\_current&ID=73894 |
|  | Reactome##113429##Elongating transcript encounters a lesion in the template##http://www.reactome.org/cgi-bin/eventbrowser?DB=gk\_current&ID=113429 |
|  | Reactome##73946##Abortive initiation##http://www.reactome.org/cgi-bin/eventbrowser?DB=gk\_current&ID=73946 |
|  | Reactome##74160##Gene Expression##http://www.reactome.org/cgi-bin/eventbrowser?DB=gk\_current&ID=74160 |
|  | KEGG pathway##00230##Purine metabolism##http://www.genome.jp/dbget-bin/show\_pathway?hsa00230+5433 |
|  | Reactome##113409##Abortive termination of early transcription elongation by DSIF##http://www.reactome.org/cgi-bin/eventbrowser?DB=gk\_current&ID=113409 |
|  | KEGG pathway##03020##RNA polymerase##http://www.genome.jp/dbget-bin/show\_pathway?hsa03020+5433 |
|  | Reactome##112386##Pausing and recovery of elongation##http://www.reactome.org/cgi-bin/eventbrowser?DB=gk\_current&ID=112386 |
|  | Reactome##75891##Abortive Initiation After Second Transition##http://www.reactome.org/cgi-bin/eventbrowser?DB=gk\_current&ID=75891 |
|  | Reactome##75856##Abortive Initiation Before Second Transition##http://www.reactome.org/cgi-bin/eventbrowser?DB=gk\_current&ID=75856 |
|  | KEGG pathway##00240##Pyrimidine metabolism##http://www.genome.jp/dbget-bin/show\_pathway?hsa00240+5433 |
|  | Reactome##75862##Fall Back to Closed Pre-initiation Complex##http://www.reactome.org/cgi-bin/eventbrowser?DB=gk\_current&ID=75862 |
|  | Reactome##74159##Transcription##http://www.reactome.org/cgi-bin/eventbrowser?DB=gk\_current&ID=74159 |


---

|  |  |
| --- | --- |
| Pathway | Zn xs inventory |
|  | Zn xs DIN |


---

|  |  |
| --- | --- |
| GO Process | transcription |
|  | transcription from RNA polymerase II promoter |


---

|  |  |
| --- | --- |
| UniGene | Mm.279898 |
|  | Hs.444887 |


---

|  |  |
| --- | --- |
| Affymetrix Probeset ID | 1424258\_at |
|  | 203664\_s\_at |
|  | 214144\_at |
|  | 243789\_at |
|  | 33726\_at |
|  | 63651\_at |
|  | 97315\_at |
|  | g4758573\_3p\_s\_at |
|  | Hs.194638.1.A1\_3p\_at |
|  | Hs.257464.0.A1\_3p\_at |
|  | Msa.14654.0\_s\_at |
|  | 228650\_at |
|  | 56881\_at |
|  | Hs.108336.0.A1\_3p\_at |
|  | TC36857\_at |
|  | U85510\_at |


---

|  |  |
| --- | --- |
| EC Number | EC 2.7.7.6 |


---

|  |  |
| --- | --- |
| GO Function | DNA-directed RNA polymerase activity |
|  | transferase activity |
|  | nucleotidyltransferase activity |


---

|  |  |
| --- | --- |
| Nucleotide | NM\_027101 |
|  | U85510 |
|  | BC093795 |
|  | BC010427 |
|  | AK011600 |
|  | BC017205 |
|  | BC093797 |
|  | AK009093 |
|  | NM\_027002 |
|  | NM\_004805 |
|  | AC006011 |
|  | AC012306 |
|  | U89387 |
|  | BC002958 |
|  | AW121730 |
|  | BC004810 |


---

|  |  |
| --- | --- |
| Protein | AAX82034 |
|  | AAH93795 |
|  | O15514 |
|  | BAB27725 |
|  | NP\_081377 |
|  | AAY14877 |
|  | AAH04810 |
|  | Q9D7M8 |
|  | NP\_081278 |
|  | NP\_004796 |
|  | AAH93797 |
|  | AAC80226 |
|  | AAC52056 |
|  | AAH17205 |
|  | AAH10427 |


---

|  |  |
| --- | --- |
| Organism | Mammal |


---

|  |  |
| --- | --- |
| Location | chromosome 2, 2q21 (Homo sapiens) |
|  | chromosome 18, 18 B2 (Mus musculus) |


---

|  |  |
| --- | --- |
